# Supramolecular Synthons in Protein–Ligand Frameworks

**DOI:** 10.1021/acs.cgd.3c01480

**Published:** 2024-02-19

**Authors:** Ronan J. Flood, Niamh M. Mockler, Aurélien Thureau, Maura Malinska, Peter B. Crowley

**Affiliations:** † SSPC, Science Foundation Ireland Research Centre for Pharmaceuticals, School of Biological and Chemical Sciences, University of Galway, University Road, Galway H91 TK33, Ireland; ‡ Synchrotron SOLEIL, L’Orme des Merisiers, Saint-Aubin BP 48, Cedex, Gif-sur-Yvette 91192, France; § Faculty of Chemistry, University of Warsaw, Pasteura 1, Warsaw 02-093, Poland

## Abstract

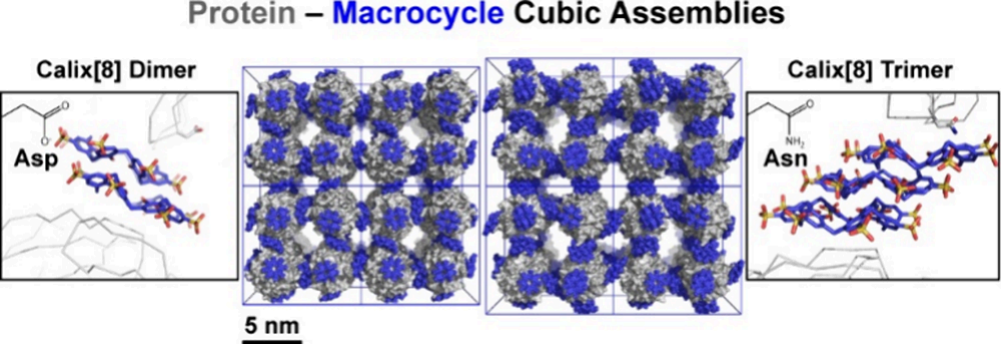

Supramolecular synthons, defined as reproducible intermolecular structural units, have greatly aided small molecule crystal engineering. In this paper, we propose that supramolecular synthons guide ligand-mediated protein crystallization. The protein RSL and the macrocycle sulfonato-calix[8]arene cocrystallize in at least four ways. One of these cocrystals is a highly porous cube comprising protein nodes connected by calixarene dimers. We show that mutating an aspartic acid to an asparagine results in two new cubic assemblies that depend also on the crystallization method. One of the new cubic arrangements is mediated by calixarene trimers and has a ∼30% increased cell volume relative to the original crystal with calixarene dimers. Crystals of the sulfonato-calix[8]arene sodium salt were obtained from buffered conditions similar to those used to grow the protein–calix[8]arene cocrystals. X-ray analysis reveals a coordination polymer of the anionic calix[8]arene and sodium cation in which the macrocycle is arranged as staggered stacks of the pleated loop conformation. Remarkably, the calixarene packing arrangement is the same in the simple salt as in the protein cocrystal. With the pleated loop conformation, the calixarene presents an extended surface for binding other calixarenes (oligomerization) as well as binding to a protein patch (biomolecular complexation). Small-angle X-ray scattering data suggest pH-dependent calixarene assembly in solution. Therefore, the calix[8]arene–calix[8]arene structural unit may be regarded as a supramolecular synthon that directs at least two types of protein assembly, suggesting applications in protein crystal engineering.

## Abstract 2

Porous cubic crystals of the RSL protein and sulfonato-calix[8]arene can be assembled via calixarene dimers or trimers. The staggered packing arrangement of the macrocycle occurs also in the sulfonato-calix[8]arene−sodium salt crystal. These results suggest that supramolecular synthons (in this case, calixarene dimers or trimers) can direct protein assembly and crystallization.

## Introduction

Designed protein assembly is a rapidly growing field with great potential in therapeutics and biotechnology.^1−7^ Porous protein-based materials, including protein cages and/or insterstices in protein crystals, are of particular interest considering their applications as biocompatible and biodegradable containers or nanoreactors. Methods to engineer protein crystals are desirable since crystallization is an important fabrication route for highly ordered materials.^4−7^ Small molecule crystal engineering has benefitted immensely from the concept of supramolecular synthons, which are intermolecular structural units that recur in many contexts.^8−10^ Here, we provide evidence for supramolecular synthons that appear to dictate protein assembly in the solid state, potentially aiding the design and fabrication processes.

Supramolecular chemistry, especially host–guest complexation, is contributing to solving the challenges of protein assembly and crystallization.^11−13^ The protein cocrystallization capacities of synthetic macrocycles such as calixarenes,^12−18^ crown ethers,^19−21^ and cucurbiturils^22−24^ have been amply demonstrated. In these structures, the crystal packing is often mediated by the macrocycle, in some cases to the exclusion of protein–protein interfaces.^13,14,16,17^ Although this work is at an early stage, there is evidence of macrocycle self-assembly that might aid protein crystal engineering. For example, variations on trimeric or tetrameric cucurbit[7]uril clusters have been obtained in cocrystals with proteins.^23,24^ Likewise, dimeric^12,16,17^ and trimeric^15^ calixarene assemblies can cocrystallize with proteins. Phosphonato-calix[6]arene (**pclx**_**6**_) dimers mediated by multiple CH-π and π–π bonds may be considered a supramolecular synthon as they mediate different crystalline frameworks.^12,16^ There are also features such as protein–calixarene interfaces (complexes) that reoccur in distinct cocrystals. The polyanionic and conformationally flexible sulfonato-calix[8]arene (**sclx**_**8**_) can mask different patches of cationic cytochrome *c* giving rise to three cocrystal forms with different symmetries and assemblies.^13,14^ Calixarene complexation of the dilysine motif K72/K73 recurs in all three structures, albeit with variations in the side chain and macrocycle conformations as well as altered protein–calixarene–protein contacts. Notably, the interaction at K72/K73 reoccurred in a ternary cocrystal of cytochrome *c*, **sclx**_**8**_, and **pclx**_**6**_.^16^

The β-propeller RSL crystallizes readily with **sclx**_**8**_, yielding at least four cocrystal forms depending on the precipitant and the pH.^17,18^ This system also includes reproducible protein–calixarene complexes that reoccur in distinct polymorphs. Two of the RSL–**sclx**_**8**_ cocrystal forms involve an essentially identical calixarene complexation with Lys25, Glu43, and Lys83. The pH is important in this system since cocrystallization below the isoelectric point of RSL (p*I* ∼ 7) enables Coulombic attraction with the macrocycle. At pH 5–6, RSL and **sclx**_**8**_ cocrystallize in space group *H*32. At pH ≤ 4, cocrystals are obtained in space groups *P*3 or *I*23. All three of the pH < p*I* crystal forms are mediated by the calixarene, devoid of protein–protein interfaces, and highly porous with a >50% solvent content. Together with NMR data, the crystal structures suggest a pH trigger enabling RSL–**sclx**_**8**_ complexation and assembly. Of the six acidic residues (3 Asp, 3 Glu) per RSL monomer, two have elevated p*K*_a_ values (Asp32 and Asp46) in the presence of **sclx**_**8**_.^17^ Calixarene complexation and protonation of these residues appear to be coupled at pH ≤ 4. The p*K*_a_ of Glu43 is unusually high (5.9, due to coplanar stacking with the indole of Trp74) and is apparently insensitive to **sclx**_**8**_.

In an effort to investigate further the role of charge, we tested three mutants in which each aspartic acid of RSL was replaced by asparagine. We hypothesized that the RSL mutants D32N and D46N would yield cocrystals with **sclx**_**8**_ at pH 5, while D77N would be unaffected. We tested cocrystallization of **sclx**_**8**_ with each RSL-DXN mutant (*X* = residue number 32, 46, or 77) in the original experimental conditions. Two of the mutants yielded the original *I*23 structure.

In contrast, one of the mutants yielded two new cubic arrangements, with altered assemblies and **sclx**_**8**_ oligomers. The occurrence of different macrocycle oligomers prompted a structural analysis of **sclx**_**8**_. Interestingly, crystals of the **sclx**_**8**_–sodium salt grow from buffered conditions similar to those used to grow the protein–calix[8]arene cocrystals. The crystal structure reveals a calixarene packing arrangement similar to that present in the protein–**sclx**_**8**_ cocrystals. Together with SAXS analysis of **sclx**_**8**_, these data suggest that a calix[8]arene–calix[8]arene supramolecular synthon dictates protein assembly. Related examples with other types of ligands are discussed.

## Materials and Methods

### Materials

Stock solutions of **sclx**_**8**_ (Tokyo Chemical Industry) were prepared in water, and pH was adjusted to 7.5. Modified pET25rsl vectors encoding RSL-D32N, RSL-D46N, and RSL-D77N were synthesized by Genscript. The proteins were produced in *Escherichia coli* BL21, purified by mannose-affinity chromatography, and prepared in 20 mM Tris-HCl, 50 mM NaCl, and 5 mM d-fructose, pH 7.5.^17^ Protein concentrations were determined spectrophotometrically with ε_280_ = 44.5 mM^–1^ cm^–1^ for the RSL monomer.

### Crystallization Trials

Crystallization was performed by hanging drop vapor diffusion in 24-well Greiner plates at 20 °C. Typical test solutions comprised 1 mM protein, 10 mM **sclx**_**8**_, and 5 mM d-fructose. Drops were prepared by mixing 1 μL of the protein–ligand mixture with 0.5 μL of reservoir solution comprising 0.5–1.5 M ammonium sulfate and 0.1 M sodium citrate pH 4.0–5.0, as described previously.^17^ Microseeding experiments^25^ were performed by including 0.5 μL of the RSL–**sclx**_**8**_*I*23 cocrystal seed stock. The seeds were generated by adding 6 μL of the reservoir (0.8 M ammonium sulfate, 0.1 M sodium citrate pH 4.0) to a <2 μL drop containing RSL–**sclx**_**8**_*I*23 crystals. The crystals were crushed into a fine suspension and combined with a further 44 μL of reservoir solution and a seed bead (Douglas Instruments) followed by four rounds of vortexing for 30 s and incubating on ice for 30 s. Cross-linking and dehydration were performed in attempts to optimize resolution. Cross-linking was performed with a Greiner CrystalBridge (Jena Bioscience) serving as a reservoir for glutaraldehyde.^26^ Crystals were incubated for 45 min prior to harvesting. Dehydration was performed by incubating crystals for 24 h against reservoir solutions containing 25–33% higher precipitant concentrations.^27^ Crystallization was also performed in bulk using ∼100 μL volumes in microcentrifuge tubes (the *P*3 condition). Crystals of the Na–**sclx**_**8**_ salt were grown in 0.6–1.6 M sodium citrate at pH 4–6 via hanging drop vapor diffusion. Crystals were imaged using an Olympus SZX16 stereomicroscope and an Olympus DP25 digital camera.

### X-ray Data Collection, Processing, and Model Building

Crystals were cryo-protected in the crystallization solution supplemented with 20–25% (v/v) glycerol and cryo-cooled in liquid nitrogen. For each crystal type, diffraction data were collected, on at least two separate occasions, at beamline PROXIMA-2A, SOLEIL synchrotron (Saint-Aubin, France) with an Eiger X 9M detector. Data were processed using the autoPROC pipeline,^28^ with integration in XDS^29^ and scaling and merging in AIMLESS^30^ and POINTLESS^31^ (Tables S1 and S2). Sulfur single-wavelength anomalous diffraction (S-SAD) data were collected at a wavelength of 2.03 Å.^17^ Substructure determination, phasing, and model building were performed in phenix.autosol^32,33^ and phenix.autobuild.^33^ Native structures were solved via molecular replacement in PHASER,^34^ using the RSL monomer (PDB 2bt9) as a search model. The coordinates for **sclx**_**8**_ (PDB id EVB) and d-fructose (PDB id BDF) were added to each model in COOT.^35^ Model building in COOT and refinement in phenix.refine^36^ were performed iteratively until no further improvements in the *R*_free_ or electron density could be made. The structures were validated in MolProbity^37^ before deposition in the Protein Data Bank with accession codes 8q6a (*I*2_1_3), 8q6b (*I*23), and 8q6c (*P*6_3_). Crystal pore diameters were calculated in MAP_CHANNELS.^38^

The structure of the Na–**sclx**_**8**_ salt crystal was solved in SHELXT^39^ and refined in SHELXL.^40^ Refinement was based on *F*^2^ for all reflections, except those with negative intensities. Weighted *R-*factors (wR) and all goodness-of-fit values (*S*) were based on *F*^2^, whereas conventional *R*-factors were based on amplitudes, with *F* set to zero for negative *F*^2^. The atomic scattering factors were obtained from the International Tables for Crystallography.^41^ Data collection and processing statistics are summarized in Table S3. The structure is available at CCDC 2298745.

### SAXS Data Collection and Analysis

SAXS data were collected at the SWING beamline (SOLEIL synchrotron) using the direct injection mode.^42^ Samples of **sclx**_**8**_ (50, 25, 12.5, 6.25, and 3.125 mM) were prepared in 0.2 M sodium citrate at pH 4 or 6. The scattering images were processed using the FOXTROT program (SOLEIL Synchrotron). Data processing included masking, azimuthal averaging, selecting and averaging curves, and subtracting the buffer signal.

## Results

### Crystal Growth and Synchrotron Data Collection

**Figure 1:**
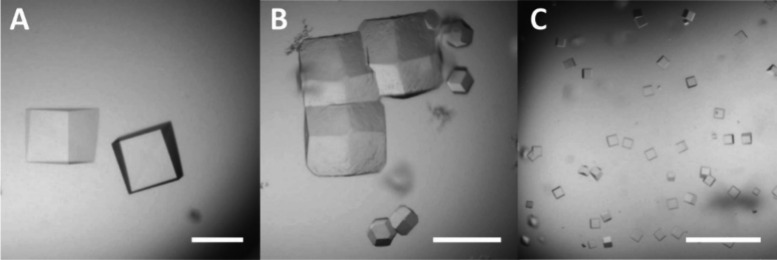
Representative cocrystals of **sclx**_**8**_ and (A) RSL, (B) RSL-D32N, and (C) microseeded RSL-D32N. The crystallization conditions comprised ∼1 M ammonium sulfate and 0.1 M sodium citrate, pH 4 at 20 °C. The scale bars are 100 μm.

The RSL–**sclx**_**8**_*I*23 cocrystals grow in ∼1 M ammonium sulfate and 0.1 M sodium citrate pH 4 at 20 °C. The *P*3 cocrystals grow without a precipitant in sodium acetate pH 4 at 4 °C.^17^ Considering the growth of these cocrystals at pH 4 and the prominent location of Asp32 in one type of RSL–**sclx**_**8**_ interface, we hypothesized that the D32N mutation would facilitate cocrystallization at pH > 4. Replacing the acidic side chain with an amide would alleviate charge repulsion with the acidic macrocycle. Cubic RSL–**sclx**_**8**_ cocrystals of ∼100 μm dimension grow within 4–5 days (Figure 1A).^17^ Similar conditions yielded RSL-D32N–**sclx**_**8**_ cocrystals with an altered morphology (Figure 1B). Small cubic crystals (Figure 1C) grew within 3–4 days when RSL-D32N crystallization drops were microseeded with RSL–**sclx**_**8**_ cubic cocrystals. Despite repeated attempts, the original cubic crystals could not be obtained with RSL-D32N. The other two mutants, RSL-D46N and RSL-D77N, yielded the original *I*23 cubes. When seeding was tested with RSL, RSL-D46N, or RSL-D77N, the original *I*23 form was again obtained in each case (Figure S1). Diffraction data were collected at SOLEIL synchrotron to 2.6 and 1.5 Å resolution for the unseeded and seeded RSL-D32N–**sclx**_**8**_ cocrystals, respectively. No significant improvement in resolution was obtained for unseeded crystals by cross-linking or dehydration. The crystal structures were solved in space groups *I*2_1_3 or *I*23 (seeded). The data for RSL-D46N or RSL-D77N–**sclx**_**8**_ cocrystals were essentially identical to the original *I*23 structure (PDB 6z5g), indicating that these mutations had a negligible effect on the assembly.

RSL–**sclx**_**8**_ cocrystals can also be obtained by incubating the protein–macrocycle mixture in sodium acetate pH 4 at 4 °C (the *P*3 condition).^17^ RSL-D32N–**sclx**_**8**_ cocrystals grew under these conditions (Figure S2) and diffracted to 1.5 Å resolution. The structure was solved in *P*6_3_ and is identical within error to the original RSL–**sclx**_**8**_ structure (PDB 6z5q).

### Altered Cubic Assemblies with RSL-D32N

**Figure 2:**
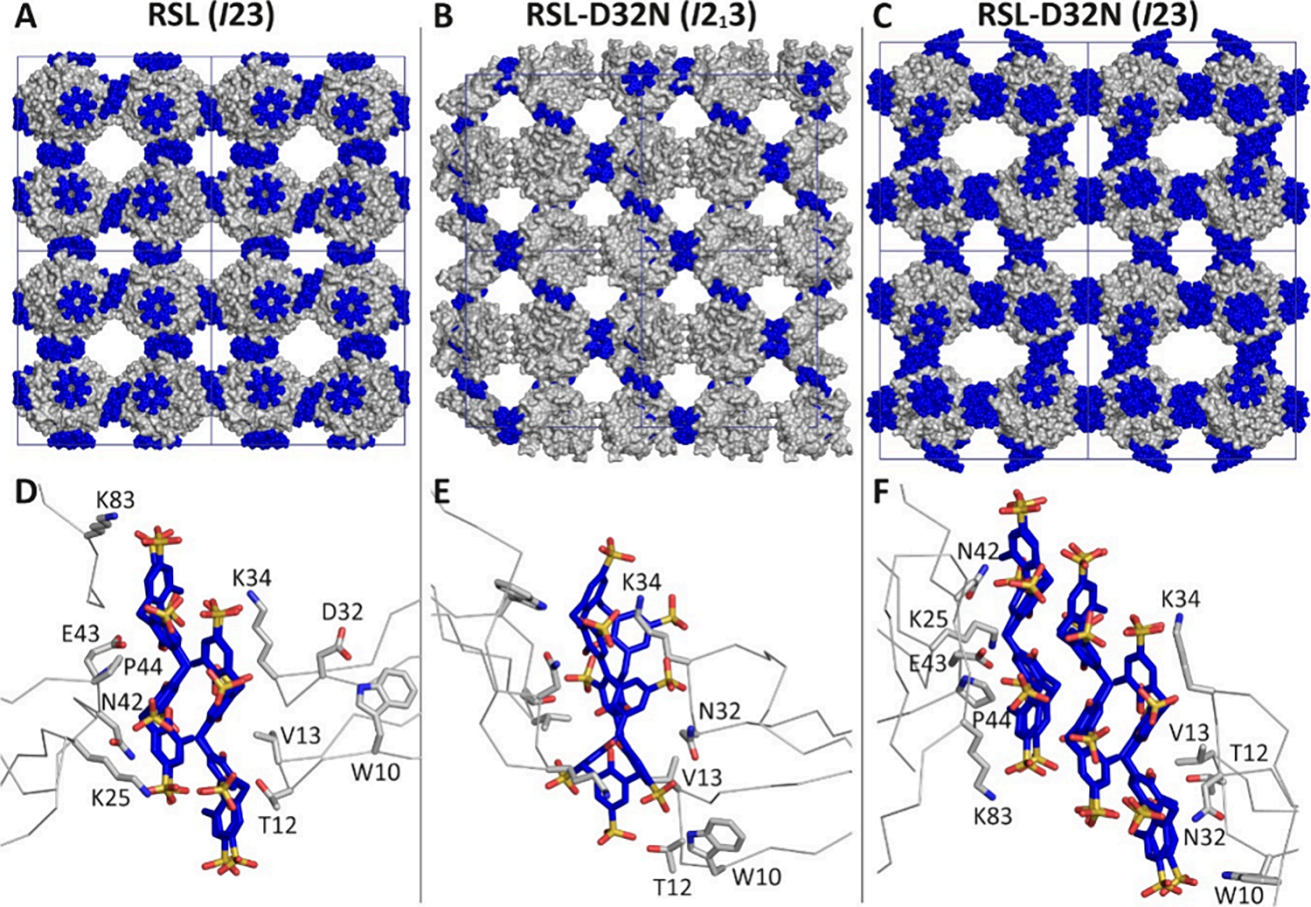
(A–C) Crystal packing in **sclx**_**8**_ cocrystals with RSL (*I*23),^17^ RSL-D32N (*I*2_1_3), and microseeded RSL-D32N (*I*23). Protein shown as gray surfaces, **sclx**_**8**_ as blue spheres, and unit cell axes in blue. (D–F) Corresponding protein–macrocycle–protein interfaces in each crystal form, containing the calixarene dimer, monomer, and trimer, respectively. RSL is shown as the monomer for clarity, with selected side chains represented as sticks. Note, the calixarene binding site in panel (E) is *C*_2_ symmetric (see also Figure S3).

The original RSL–**sclx**_**8**_*I*23 cocrystals are a cubic assembly, in which one protein node is connected to six others via calixarene dimers (Figure 2).^17^ There are no protein–protein interfaces in this structure. Rather, the crystal packing junctions comprise one calixarene–protein interface involving Val13 and Lys34, a calixarene–calixarene interface, and a second calixarene–protein interface involving Lys25, Glu43, and Lys83. The RSL-D32N mutant yields an altered assembly, in space group *I*2_1_3, with a single calixarene at a special position (crystallographic 2-fold axis), mediating a *C*_2_-symmetric junction at Val13 and Lys34 (Figure 2). While the calixarene is evident in the unbiased electron density map (Figure S3), the model building was challenging due to the poor resolution and the *C*_2_ symmetry. S-SAD data confirmed the presence of the calixarene and aided the model building (Figure S4) with two symmetry-related calixarenes at 50% occupancy. An alternative model using half of a calixarene resulted in unmodeled density, corresponding to two units of the macrocycle (Figure S3), and was discarded. The calixarene has a distorted conformation, similar to that in the RSL–**sclx**_**8**_*P*3 crystal form,^17^ and masks about 440 Å^2^ of the protein surface. Surprisingly, the Lys25/Lys83 patch of RSL is devoid of calixarene (confirmed by S-SAD; Figure S4) and adjacent proteins in the crystal packing are separated by the van der Waals distance (∼4.5 Å). The occurrence of such “noncontact” protein–protein junctions might be a contributing factor to the poor resolution. Despite repeated attempts to improve resolution, testing both large and small crystals (Figure 1B) as well as cross-linking or dehydration, the maximum resolution obtained from this crystal form was 2.6 Å. This poor resolution was attributed to disorder in the crystal arising from the incomplete crystal packing at the Lys25/Lys83 patch.

The seeded RSL-D32N–**sclx**_**8**_ cocrystals were solved in space group *I*23. Here, the assembly is similar to the original RSL–**sclx**_**8**_*I*23 structure but the protein nodes are connected via calixarene trimers instead of dimers (Figure 2 and Figure S5). Of the two types of protein–calixarene interface, the **sclx**_**8**_ complex at Lys25/Glu43/Lys83 is identical within error to that in the RSL–**sclx**_**8**_*I*23 and *H*32 structures.^17,18^ In contrast, the protein–calixarene interface at Lys34 is markedly altered. While Val13 and Lys34 remain key interface residues, the calixarene has moved ∼1 nm along the protein surface such that it also masks the side chain of Asn32. This binding mode is similar to that in the RSL–**sclx**_**8**_*P*3 crystal form.^17^ While the calixarene trimer-mediated form was obtained by seeding, it did not occur with native RSL, suggesting that both the replacement of the acidic side chain by the amide and relocation of the complexing calixarene enabled the assembly. This crystal form was obtained at 5–20 mM **sclx**_**8**_, suggesting that the calixarene concentration does not determine oligomerization. With three calixarenes per protein monomer, the macrocycle:protein mass ratio is approximately 1:2, yielding a hybrid material.^23^ The insertion of an additional calixarene into the crystal packing results in an expansion of the unit cell dimension, from 104 to 112 Å, and a corresponding ∼1.3-fold volume increase (Table 1). In contrast, the *I*2_1_3 assembly with a single calixarene has a decreased unit cell dimension of 95 Å and an ∼1.3-fold volume decrease. The unit cell volume changes have concomitant porosity changes, as indicated by two types of calculation (Table 1).

**Table 1:** Parameters of RSL–**sclx**_**8**_ Cubic Cocrystal Forms

**protein**	**PDB**	**space group**	*a***×***b***×***c***(Å)**	**res. (Å)**	**ratio**^a^	**S.C.(%)**^b^	**pore** Ø **(nm)**^c^	**cell volume variation**
RSL	6z5g	*I*23	104^3^	1.3	1:2	66	4.2	
RSL-D32N	8q6a	*I*2_1_3	95^3^	2.6	1:1	61	2.9	∼1.3× decrease
RSL-D32N	8q6b	*I*23	112^3^	1.5	1:3	70	5.4	∼1.3× increase

a Ratio of RSL monomer:**sclx**_**8**_ in the asymmetric unit.

b Solvent content (Matthews coefficient) estimated from total mass (protein plus **sclx**_**8**_).

c Diameter of the widest pore, calculated in MAP_CHANNELS.

### Calixarene Self-Assembly

Considering the occurrence of **sclx**_**8**_ dimers and trimers in protein cocrystals (Figure 2), we attempted to assess the contribution of calixarene self-assembly to the cocrystallization process. To date, at least 20 solid state structures of **sclx**_**8**_ have been reported (Table S4).^43−54^ With one exception, these structures include cationic guests that influence the conformation and crystal packing of the calixarene host. **sclx**_**8**_ can adopt regular conformations such as the pleated loop or the inverted double cone in complex with the butanediammonium or the cyclohexyldiammonium cation, respectively.^44^ Highly distorted conformations occur when the calixarene wraps around bulky guests such as cobalt-tris(phenanthroline)^45^ or phenanthroline oligomers.^50^ Recently, Danylyuk and co-workers reported crystals that were obtained by slow diffusion of ethanol into an aqueous solution of the **sclx**_**8**_ sodium salt.^54^ In this structure, **sclx**_**8**_ is in the pleated loop conformation and forms columnar stacks. The stacks are almost perfectly superposed calixarenes, yielding tubes with a solvent-filled core and an outer layer of sodium cations coordinating the sulfonates. The pleated loop conformation is a low-energy state (see also *t*-butyl-calix[8]arene^55^) as the sulfonate groups are as far apart as possible, thus minimizing Coulombic repulsion (Table S5).

**Figure 3:**
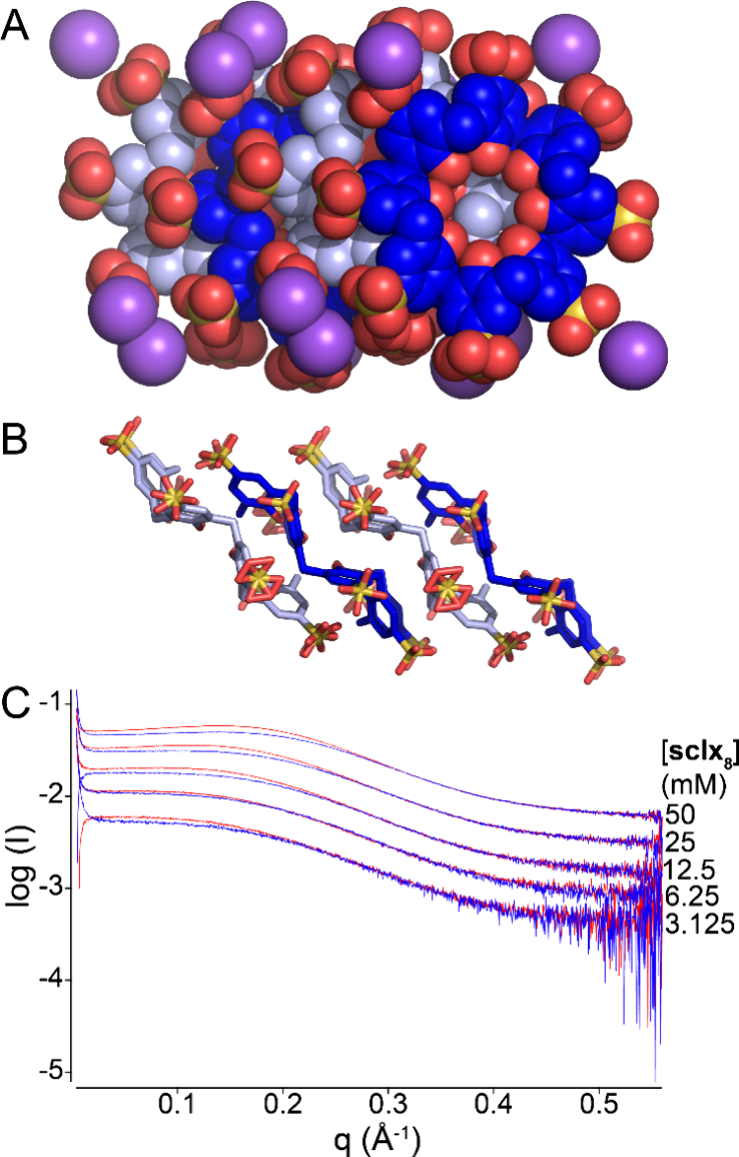
Crystal packing in a Na–**sclx**_**8**_ salt structure with calixarene represented as (A) spheres or (B) sticks. Sodium ions are purple. Waters are omitted for clarity. (C) Logarithmic SAXS profiles of **sclx**_**8**_ at varying concentrations (3–50 mM) in 0.2 M sodium citrate pH 4 (red) or pH 6 (blue).

Previously, rod-shaped crystals or needles of **sclx**_**8**_ were obtained via hanging drop vapor diffusion of RSL–calixarene mixtures in sodium citrate pH 4–6.^18^ The crystals were reproduced in the absence of protein at 2–30 mM **sclx**_**8**_ (Figure S6). Crystals grew in 1–3 days, and the concentration of citrate required for crystallization decreased with decreasing pH. While crystallization at pH 6 required >1.4 M sodium citrate, at pH 4, crystals were obtained at ≥0.6 M sodium citrate. The crystal structure was solved using data collected to 0.79 Å resolution at SOLEIL synchrotron. This structure again involves the pleated loop conformation. However, the packing is staggered, with adjacent pairs of calixarenes forming multiple CH-π, π–π, and anion-π bonds (Figure 3). Within a trimeric stack of **sclx**_**8**_, there are also four pairs of repulsive anion–anion interactions. A similar conformation and packing arrangement are possible with *t*-butyl-calix[8]arene, in which the *tert*-butyl substituents and the phenol units form CH-π bonds.^55^ The staggered (or staircase) packing in the Na–**sclx**_**8**_ salt (Figure 3B) is similar to the dimer and trimer calixarene arrangements observed in the protein–**sclx**_**8**_ cocrystals (Figure 2F).

In addition to the solid state study, we collected small-angle X-ray scattering (SAXS) data of **sclx**_**8**_ (3–50 mM) in 0.2 M sodium citrate at pH 4 or 6. Figure 3C shows the scattering curves. The maximum intensity shifts to higher *q* values with increasing sample concentration, indicative of increasing particle sizes. This effect is more pronounced at pH 4 where Na–**sclx**_**8**_ salt crystallization occurs more readily. These data are consistent with **sclx**_**8**_ assembly in solution and hint at an explanation for protein and **sclx**_**8**_ dimer/trimer cocrystallization at pH 4 only.

## Discussion

This work was inspired by the pH dependence of RSL–**sclx**_**8**_ cocrystallization. Formation of the *P*3 cocrystal (PDB 6z5q) at pH 4 and the prominence of Asp32 in the protein–calixarene interface, together with NMR data, suggest that the Asp32 side chain is protonated upon calixarene complexation.^17^ Asp46 is also located close to the calixarene in this structure. Therefore, we hypothesized that replacing the acidic side chain with an amide would favor crystal growth at pH 5 or higher. This hypothesis proved to be false as the D32N mutation had minimal effect on the *P*3 crystallization. Similarly, the mutants D46N and D77N had no significant effects on protein–**sclx**_**8**_ cocrystallization.

The *I*23 cocrystal form (PDB 6z5g) also requires pH 4. In this structure, the Asp32 side chain is ∼8 Å distant from the calixarene. Crystallization of RSL-D32N using the original conditions^17^ yielded a new crystal form (space group *I*2_1_3; Figure 2B) with a *C*_2_ symmetric protein–calixarene–protein interface involving Asn32 and Lys34 (Figure 2E). Apparently, replacing the acidic side chain and the consequent increase in cationic character facilitated the involvement of this protein patch in calixarene complexation and crystal growth. The new assembly is depleted of a protein–calixarene–protein interface at the Lys25/Lys83 patch. Poor packing at this site may have contributed to an overall increase in disorder for this structure (and hence the 2.6 Å resolution). This result is also interesting in that a *C*_2_ symmetric protein–calixarene–protein interface at Lys25/Lys83 occurs in the *H*32 crystal form.^18^

The combination of RSL-D32N with RSL–**sclx**_**8**_*I*23 cocrystal seeds resulted in another new crystal form. In this case, the space group was preserved but the original assembly involving a calixarene dimer was augmented to a calixarene trimer (Figure 2F). The relative orientation of the protein nodes is similar in the original (RSL) and the new (RSL-D32N) *I*23 crystal forms. In the latter, the unit cell volume has increased to accommodate the insertion of an extra calixarene. These results point to the possibility of protein crystal engineering by macrocycle oligomerization.

The pleated loop conformation of **sclx**_**8**_ was demonstrated crystallographically as early as 2006 in a cocrystal with butanediammmonium cations.^44^ The first protein–**sclx**_**8**_ cocrystal structures were reported in 2018, some of which contained pleated looplike conformations.^13^ The regular pleated loop **sclx**_**8**_ was reported in 2021 in a protein cocrystal (PDB 6z5g)^17^ and in a sodium salt structure (CCDC XEXZAF).^54^ Thus, this conformation can occur with either small molecule or macromolecule guests as well as in the absence of a guest. In the course of RSL–**sclx**_**8**_ cocrystallization trials, we observed that sodium citrate pH 4–6 leads to calixarene crystallization.^18^ In this sodium salt structure (CCDC 2298745), the calixarene is again in the pleated loop conformation and has a staggered packing arrangement (Figure 3). The **sclx**_**8**_ dimer and trimer assemblies in protein cocrystals (Figure 2) are essentially identical to the structural arrangement of **sclx**_**8**_ in the sodium salt. These observations suggest that the calixarene oligomer, favored by sodium citrate pH 4, enables RSL–**sclx**_**8**_ cocrystallization. SAXS data provide further evidence for increased **sclx**_**8**_ assembly in sodium citrate at pH 4, compared to pH 6. Cocrystallization of the calixarene trimer with RSL-D32N appears to require both the mutation and the presence of microseeds but is not dependent on the calixarene concentration (in the 5–20 mM range). Indeed, the original *I*23 structure, with calixarene dimers, was also obtained at 5–50 mM **sclx**_**8**_. Thus, similar crystallization conditions give rise to interfaces comprising one, two, or three calixarenes. It is notable that crystals of the Na–**sclx**_**8**_ salt were obtained with 2–30 mM macrocycle under similarly buffered conditions.

With the increasing number of protein–macrocycle cocrystal structures,^11−24^ evidence is accumulating in favor of supramolecular synthons that direct assembly and framework fabrication. The RSL–**sclx**_**8**_ synthon with the pleated loop calixarene bound to the protein at Lys25/Glu43/Lys83 occurs in three distinct cocrystals (Figure 2 and PDBs 6z5g, 8c9z, and 8q6b).^17,18^ More importantly, the same calix[8]arene–calix[8]arene synthon appears as a dimer or trimer in cocrystals with proteins (Figure 2) and as extended stacks in the sodium salt crystal (Figure 3). Cucurbit[7]uril clusters^23^ and dimeric or trimeric stacks of tetra(4-sulfonatophenyl)porphyrin^56^ have also been reported in protein cocrystals. Together, these observations suggest that macrocycle oligomerization^57,58^ can direct protein assembly. Here, it is insightful to compare with protein–foldamer assemblies. Quinoline-based foldamers form highly defined supramolecular synthons that can mediate protein dimerization (PDB 5lvs).^59^ In a cocrystal structure with cytochrome *c*, a tetrameric π–π stacked foldamer dominated the crystal packing (PDB 6s8y).^60,61^ π–π stacking has been observed also in protein cocrystals with small molecule aromatic ligands. The acetylcholine-binding protein can accommodate π–π stacked dimers or trimers of an isoquinoline-containing drug candidate (PDB 4bfq).^62^ Recently, a stunning example of π–π stacking was reported with ubiquitin-specific protease USP15 in complex with the anthracenedione mitoxantrone (PDB 7r2g).^63^ The latter forms an ∼4 nm long dodecameric stack, which, like the foldamer example,^61^ dominates the crystal packing. A similar π–π stack is found in crystals of the parent molecule 1,4-diamino-9,10-anthraquinone (CCDC EQAHEL).^64,65^ Guided protein assembly via π–π ligand stacking has been achieved also with lectins bound to sugar-rhodamine conjugates, in which rhodamine dimerization appears to direct the process.^66^

## Conclusions

Previously, we reported an extended arm calixarene that assembles as trimers when cocrystallized with cytochrome *c*.^15^ This macrocycle assembly has four grooves, each of which can accommodate one α-helix of a protein. While these calixarene oligomers were necessary for cocrystallization, the mode of protein complexation was substantially different with respect to **sclx**_**8**_. The present work, building on a previous example with dimers,^17^ firmly establishes the possibility of **sclx**_**8**_ trimers as mediators of protein assembly in the solid state. With the pleated loop conformation, the calixarene presents an extended surface for binding other calixarenes (oligomerization) as well as binding to a protein patch (biomolecular complexation). With a 2:1 protein:macrocycle mass ratio, the RSL-D32N–**sclx**_**8**_ assembly is a hybrid material (Figure 2C). Together with other studies,^13−18^ it is evident that protein–**sclx**_**8**_–protein crystal packing junctions can occur with the macrocycle as a monomer, dimer, or trimer. Numerous cocrystal structures of **sclx**_**8**_ with small molecules have revealed the broad range of conformations that the macrocycle uses to accommodate different guests (Table S4). However, none of the small molecule cocrystal structures involve macrocycle oligomers. Interestingly, two Na–**sclx**_**8**_ salt structures are now available. In both structures, the calixarene occurs as oligomeric stacks, assembled from the regular pleated loop conformation. This conformation is amenable to columnar stacking (with a central solvent channel)^54^ or staggered stacking (Figure 3A). The latter arrangement is essentially identical to that found in the protein cocrystal (Figure 2D,F). We conclude that a supramolecular synthons, in this case, staggered stacks of **sclx**_**8**_, are a determining factor in protein assembly. It remains to be seen if larger oligomers of the macrocycle can also form stable assemblies within protein cocrystals, paving the way to new types of hybrid materials. Future work may reveal the oligomerization and nucleation processes^67,68^ and provide insight into the steps involved in crystallization and framework fabrication.

## Supporting Information Available

X-ray crystallography statistics and electron density maps. Optical microscopy images of crystals. Tables of **sclx**_**8**_ crystal structures and calculated conformation energies (PDF)
