# Supplementary material for: Supramolecular Synthons in Protein–Ligand Frameworks
Source: Cryst Growth Des. 2024 Feb 19;24(5):2149–56. doi: 10.1021/acs.cgd.3c01480 (PMC10921380; doi:10.1021/acs.cgd.3c01480)
Supplement: Supplementary file 1 — cg3c01480_si_001.pdf [file cg3c01480_si_001.pdf]

Supporting Information

Supramolecular Synthons in Protein – Ligand Frameworks

Ronan J. Flood,[a] Niamh M. Mockler,[a] Aurélien Thureau,[b] Maura Malińska,[c] and Peter B. Crowley*,[a]

[a]SSPC, Science Foundation Ireland Research Centre for Pharmaceuticals, School of Biological and Chemical Sciences, University of Galway, University Road, Galway H91 TK33, Ireland.

[b]Synchrotron SOLEIL, L'Orme des Merisiers, Saint-Aubin BP 48, 91192 Gif-sur-Yvette Cedex, France

[c]Faculty of Chemistry, University of Warsaw, Pasteura 1, Warsaw, Poland

*Correspondence to: peter.crowley@nuigalway.ie +353 91 49 24 80

Keywords: β-propeller; biomaterials; crystal engineering; molecular glue; self-assembly.

Table S1. X-ray data collection, processing and refinement statistics for RSL-D32N – **sclx₈** co-crystals.

Structure	D32N – **sclx8** seeded	D32N – **sclx8** unseeded	D32N – **sclx8** unseeded SSAD
Crystallization conditions			
Protein (mM)	1	1	1
**sclx8** (mM)	10	10	10
$(NH_4)_2SO_4$ (M)	1	0.8	0.8
Buffer	0.1 M sodium citrate pH 4.0		
Data collection[a]			
**Light source**	SOLEIL, PROXIMA-2A		
**Wavelength (Å)**	0.98011		2.03253
**Space group**	*I*23	$I2_13$	$I2_13$
**Cell constants (Å)**	112.160^3	94.402^3	94.893^3
**Resolution (Å)**	45.79-1.52 (1.55-1.52)	19.27-2.61 (2.66-2.61)	47.45-2.87 (2.92-2.87)
**# reflections**	1245931 (57048)	169531 (7840)	763776 (30637)
**# unique reflections**	36271 (1805)	4376 (208)	3370 (167)
**Multiplicity**	34.4 (31.6)	38.7 (37.7)	226.6 (183.5)
**I/σ (I)**	22.6 (2.2)	15.8 (2.5)	87.0 (2.2)
**Completeness (%)**	100.0 (100.0)	100.0 (100.0)	100.0 (100.0)
R_{meas}[b]**(%)**	11.7 (203.5)	16.4 (168.3)	7.0 (287.2)
R_{pim}[c]**(%)**	2.0 (36.1)	2.6 (27.1)	0.5 (27.3)
$CC_{1/2}$	100.0 (74.8)	99.8 (84.6)	100.0 (86.4)
**Solvent content (%)**	70	60	60
Refinement			
R_{work}	17.7	21.5	
R_{free}	18.3	23.1	
**rmsd bonds (Å)**	0.005	0.003	
**rmsd angles (°)**	0.764	0.623	
**# molecules in asymmetric unit**			
**Protein chains**	1	1	
**sclx8**	3	1	
**fructose**	2	1	
**glycerol**	2	1	
**water**	65	9	
**Avg. B-factor (Å²)**	20.47	64.81	
**Clashscore**	0.6	6.7	
**Ramachandran analysis,[d] % residues in**			
**favoured regions**	96.59	94.32	
**allowed regions**	3.41	5.68	
**PDB code**	8q6b	8q6a	

[a]Values in parentheses correspond to the highest resolution shell [b]Rmeas = ∑hkl √(n/n-1)∑I |Ii(hkl)-⟨I(hkl)⟩ |/∑hkl ∑i Ii(hkl); [c]Rpim = ∑hkl √(1/n-1)∑ n i=1 |Ii(hkl)- |/∑hkl ∑i Ii(hkl); [d] ⟨I(hkl)⟩ Calculated in MolProbity.

Table S2. X-ray data collection, processing and refinement statistics for the $P6_3$ RSL-D32N – **sclx$_8$** co-crystal.

Crystallization conditions	
Protein (mM)	0.8
**sclx$_8$** (mM)	15
Buffer	20 mM sodium acetate pH 4
Data collection	
**Light source**	SOLEIL, PROXIMA-2A
**Wavelength (Å)**	0.98011
**Space group**	$P6_3$
**Cell constants (Å)**	59.82, 59.82, 64.68
**Resolution (Å)**	51.81-1.38 (1.41-1.38)
**# reflections**	398152 (20315)
**# unique reflections**	26994 (1327)
**Multiplicity**	14.7 (15.3)
**I/σ (I)**	16.0 (2.1)
**Completeness (%)**	100.0 (100.0)
**R_{meas}[b](%)**	7.9 (126.9)
**R_{pim}[c](%)**	2.1 (32.3)
**$CC_{1/2}$**	99.9 (88.4)
**Solvent content (%)**	59
Refinement	
**R_{work}**	19.5
**R_{free}**	19.6
**rmsd bonds (Å)**	0.005
**rmsd angles (°)**	0.756
**# molecules in asymmetric unit**	
**Protein chains**	1
**sclx$_8$**	1
**Fructose**	1
**Glycerol**	1
**Water**	58
**Avg. B-factor (Å^2)**	20.03
**Clashscore**	0.00
**Ramachandran analysis,[d] % residues in**	
**favoured regions**	97.73
**allowed regions**	2.27
**PDB code**	8q6c

Table S3. X-ray data collection, processing and refinement statistics for the **sclx$_8$** sodium salt.

*Crystallization conditions**	
**sclx$_8$** (mM)	32
Precipitant	1 M sodium citrate pH 5.2
Additional components	1 mM RSL, 5 mM D-fructose
Crystal data	
Chemical formula	$C_{56}H_{40}O_{53}S_8 \cdot Na_8\ O_8$
M_r (Da)	2129.28
Crystal system, space group	Monoclinic, *C*2/*c*
Temperature (K)	100
a, *b*, *c* (Å)	17.629 (4), 41.370 (8), 12.475 (3)
β (°)	91.93 (3)
V (Å^3)	9093 (3)
Z	4
Radiation type	SOLEIL Synchrotron, λ = 0.72932 Å
μ (mm^{-1})	0.37
Crystal size (mm)	0.05 × 0.05 × 0.05
Data collection	
Absorption correction	–
No. of measured, independent and observed [$I > 2\sigma(I)$] reflections	8498, 8498, 7201
R_{int}	0.028
$(\sin \theta/\lambda)_{max}$ (Å^{-1})	0.637
Refinement	
$R[F^2 > 2\sigma(F^2)]$, $wR(F^2)$, S	0.158, 0.468, 2.29
No. of reflections	8498
No. of parameters	709
No. of restraints	25
H-atom treatment	H-atom parameters constrained
$\Delta\rho_{max}$, $\Delta\rho_{min}$ (e.Å^{-3})	1.71, -1.54
CCDC deposition #	2298745

*The crystal used for structure determination was grown from a solution containing protein. The same crystal form is obtained in the absence of protein (Figure S6).

Table S4. Structures of **sclx$_8$** in the Cambridge Crystallographic Data Centre.

#	Identifier	"Guests"	Conformation[a]	Ref
1	JAPQIB	(4,4′-dipyridine-N,N′-dioxide)-Europium aqua complex	collapsed pleated loop	1
2	VERBEA	*cis*-1,2-diaminocyclohexane	inverted double cone[b]	2
3	VERBAW	1,4-Diaminobutane	**perfect pleated loop**	
4	PEJNUO	tris(phenanthroline)-Cobalt, Ytterbium aqua complex	chalice-like	3
5	DIZPEI	dimethylamine, dimethylformamide	distorted, self-encapsulated	4
6	LUKBUP	bis(4,4′-dimethyl-2,2′-bipyridine)-Nickel aqua complex	inverted double cone	5
7	QULJUD	bis(phenanthroline)-Copper complex	inverted double cone	6
8	QULKAK	bis(phenanthroline)-Zinc complex	inverted double cone	
9	HIZTUH	1,2-di(4-pyridyl)-ethane	double cone	7
10	HIZVAP	1,3-di(4-pyridyl)-propane	distorted pleated loop, 2 up–down	
11	UYUDID	phenanthroline oligomers	highly distorted, self-encapsulated	8
12	UYUDOJ	phenanthroline oligomers, Ethanol	shallow inverted double cone	
13	UYUDUP	phenanthroline oligomers		
14	LEHPUM	lidocaine *exo*	collapsed pleated loop	9
15	LEHQEX	lidocaine *endo*	inverted double cone	
16	LEHQAT	proparacaine	inverted double cone	
17	ZIYSEI	(4,4′-bipyridine-N,N′-dioxide)-Copper aqua complex	distorted pleated loop	10
18	QUCTIU	trimethylanilinium-porphyrin, 2-methyl-2,4-pentanediol	shallow treble cone	11
19	XEXVAB	berberine oligomer	distorted loop, self-encapsulated	12
20	XEXZAF	sodium salt	**perfect pleated loop**	
21	2298745	sodium salt	**perfect pleated loop**	-

[a] C_2 symmetric, except JAPQIB, UYUDID/UYUDUP, and QUCTIU.

[b] At least 3 types of inverted double cone can be distinguished.

Table S5. Calculated conformational energies of the **sclx$_8$** octa-anion.*

Conformation	Vacuum (Ha)	Water (Ha)	Vacuum (kJ/mol)	Water (kJ/mol)
Pleated loop[a]	-7747.78	-7749.81	-20341775.5	-20347113.2
Inverted double cone[b]	-7747.77	-7749.79	-20341756.3	-20347073.8
**ΔE**	0.01	0.02	19.2	39.4

*The initial **sclx$_8$** geometry, extracted from crystal structures, was optimized using the DFT method (M06-2X), within Gaussian16, employing a 6-31d basis set and the Polarizable Continuum Model.[13,14] The calculations were performed in vacuum, or in water by using the integral equation formalism variant.

[a] Coordinates derived from the Na–**sclx$_8$** crystal structure (2298745) presented in this study.

[b] Coordinates derived from CCDC VERBEA.

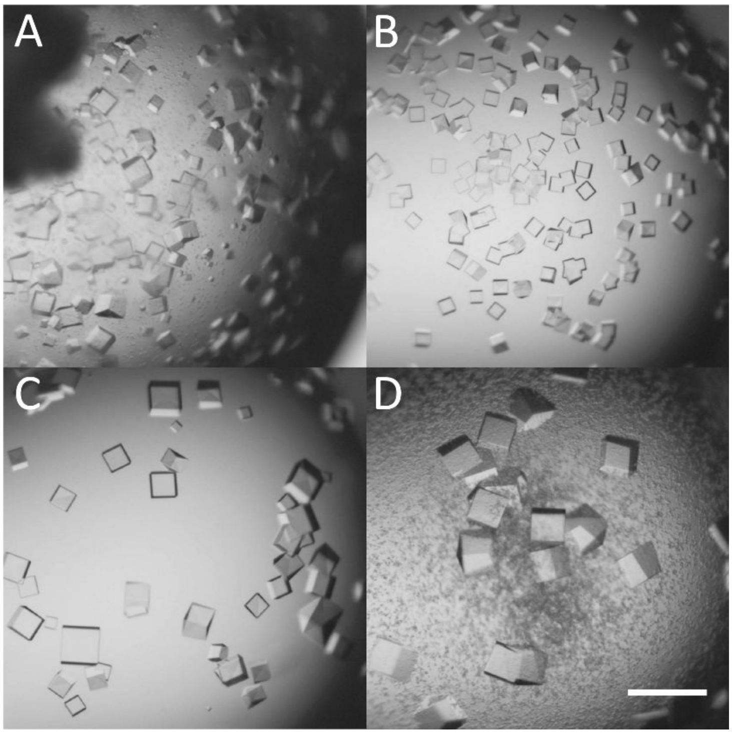

Figure S1. Co-crystals of **sclx$_8$** and **(A)** RSL-D46N without and **(B)** with micro-seeding or **(C)** RSL-D77N without and **(D)** with micro-seeding. The reservoir contained 0.8 M ammonium sulfate and 0.1 M sodium citrate pH 4. The scale bar is 100 µm.

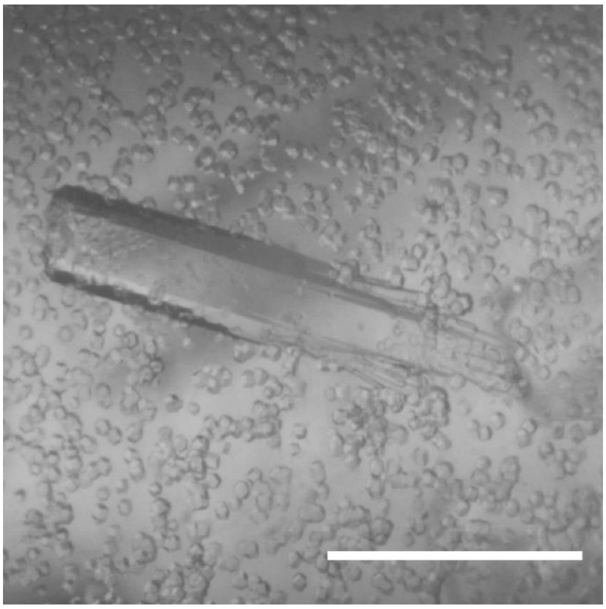

Figure S2. RSL-D32N – **sclx$_8$** co-crystals obtained, in the absence of precipitant, at pH 4 and 4 °C. The scale bar is 500 µm.

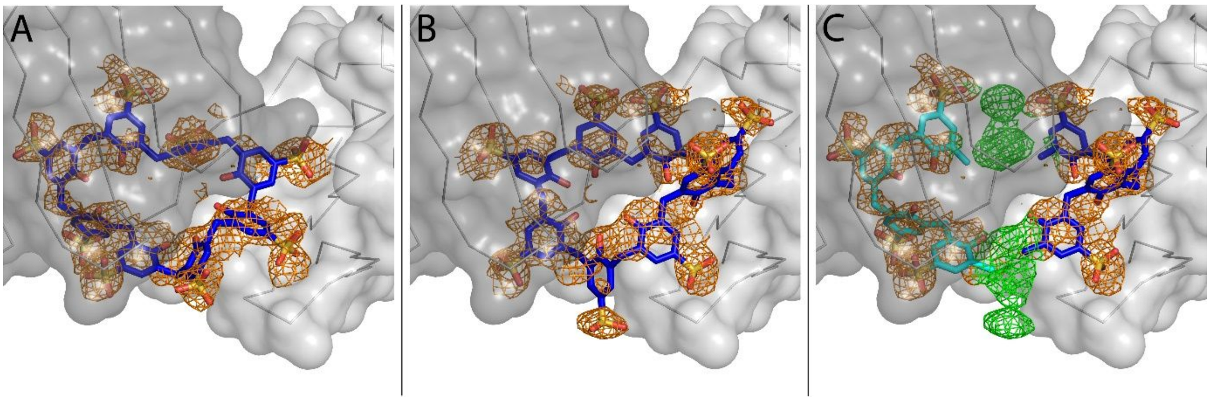

Figure S3. The unbiased 2Fo - Fc electron density map contoured at 1.0 σ (orange mesh), corresponding to calixarene at a special position (crystallographic two-fold axis) in the RSL-D32N – **sclx$_8$** $I2_13$ crystal form. Two RSL monomers are shown as light and dark grey transparent surfaces. Also shown are the refined models of **(A) sclx$_8$** at 50% occupancy and **(B)** the symmetry mate. **(C)** An alternative model built with a half calixarene (dark blue, and symmetry mate in cyan) is incomplete with unmodelled density for two **sclx$_8$** monomers (highlighted green).

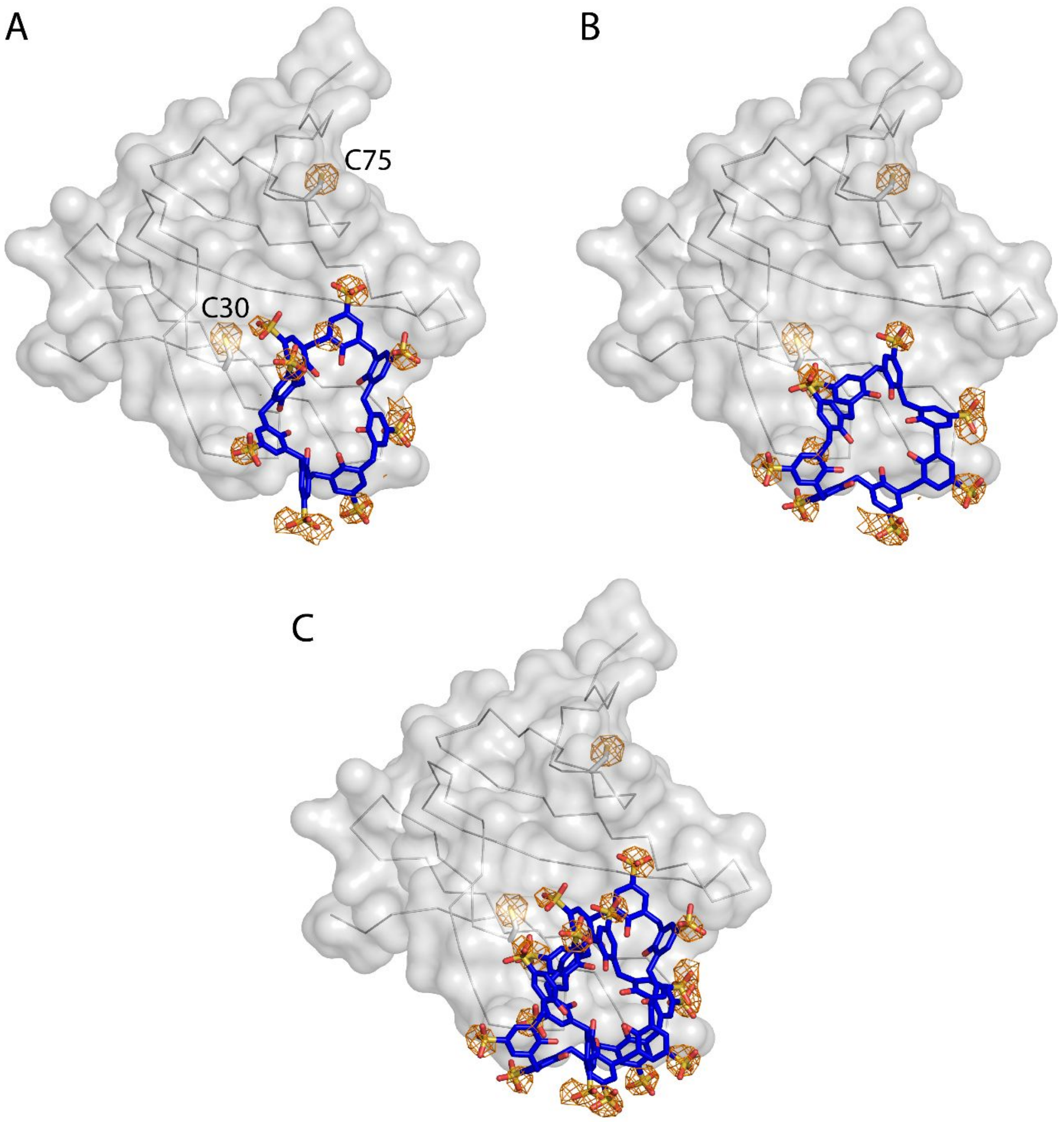

Figure S4. Fourier maps (orange mesh), calculated from sulfur single-wavelength anomalous diffraction (SSAD) data and contoured at 4.0 σ reveal the locations of the calixarene and two cysteines. Also shown are the refined models of **(A)** $\mathbf{sclx_8}$ at 50% occupancy, **(B)** the symmetry mate and **(C)** the superposed components. The RSL-D32N monomer is shown as a transparent surface and C^{α} trace, the $\mathbf{sclx_8}$ molecules and cysteine side chains are shown as sticks.

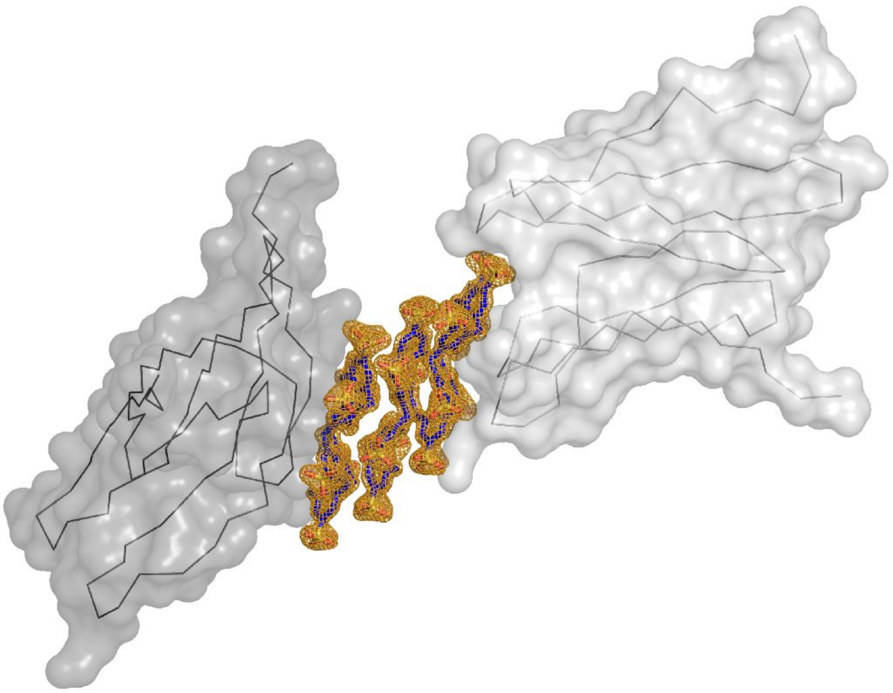

Figure S5. In the RSL-D32N – $\mathbf{sclx_8}$ *I*23 structure the presence of the trimeric $\mathbf{sclx_8}$ stack was clear in the unbiased 2Fo - Fc electron density maps, contoured at 1.0 σ (orange mesh). Two RSL monomers are shown.

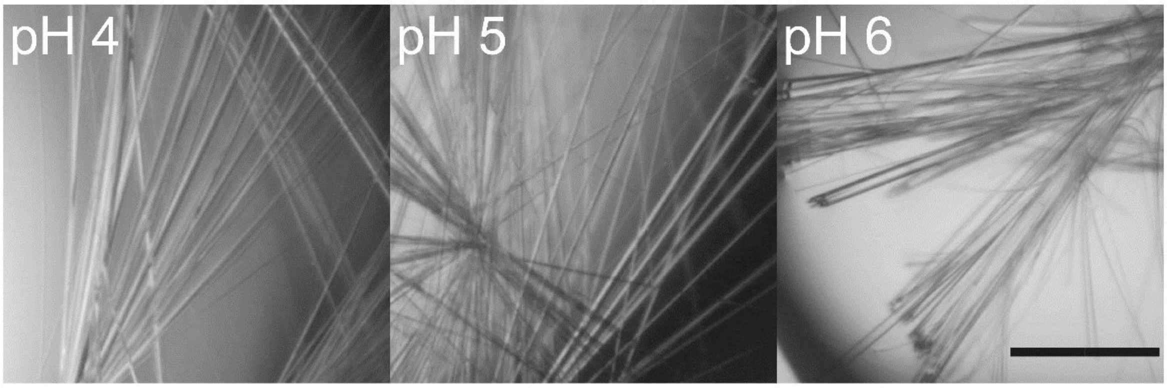

Figure S6. Crystals of Na–**sclx$_8$** salt obtained by hanging drop vapour diffusion against solutions of sodium citrate at pH 4-6 and 20 °C. The scale bar is 200 µm.

References

1. Dalgarno, S. J.; Hardie, M. J.; Atwood, J. L.; Warren, J. E.; Raston, C. L. A complex 3D 'wavy brick wall' coordination polymer based on *p*-sulfonatocalix[8]arene. *New J. Chem.* **2005**, *29*, 649-652.
2. Perret, F.; Bonnard, V.; Danylyuk, O.; Suwińska, K.; Coleman, A. W. Conformational extremes in the supramolecular assemblies of *para*-sulfonato-calix[8]arene. *New J. Chem.* **2006**, *30*, 987-990.
3. Smith, C. B.; Barbour, L. J.; Makha, M.; Raston, C. L.; Sobolev, A. N. Unlocking the elusive binding cavity in *p*-sulfonatocalix[8]arene. *New J. Chem.* **2006**, *30*, 991-996.
4. Danylyuk, O.; Perret, F.; Coleman, A. W.; Suwińska, K. The solid-state complex of *para*-sulphonato-calix[8]arene anion with dimethylammonium cations. *Open Crystallog. J.* **2008**, *1*, 18-23.
5. He, W.; Bi, Y.; Liao, W.; Li, D. A ternary supramolecular compound of *p*-sulfonatocalix[8]arene with 1D channels. *J. Mol. Struct.* **2009**, *937*, 95-99.
6. Liu, Y.; Liao, W.; Bi, Y.; Wang, M.; Wu, Z.; Wang, X.; Su, Z.; Zhang, H. 1,2,3,4-Alternate double cone conformational extreme in the supramolecular assemblies of *p*-sulfonatocalix[8]arene. *CrystEngComm* **2009**, *11*, 1803-1806.
7. Leśniewska, B.; Perret, F.; Suwińska, K.; Coleman, A. W. Structural characterization of inclusion complexes of *para*-sulphonato-calix[8]arene with 1,2-bis(4-pyridyl)-ethane and 1,3-bis(4-pyridyl)-propane. New 'double cone' and 'up–flat–down' conformations of *para*-sulphonato-calix[8]arene. *CrystEngComm* **2014**, *16*, 4399-4405.
8. Leśniewska, B.; Coleman, A. W.; Tauran, Y.; Perret, F.; Suwińska, K. Pseudopolymorphs – a variety of self-organization of *para*-sulphonato-calix[8]arene and phenanthroline in the solid state. *CrystEngComm* **2016**, *18*, 8858-8870.
9. Danylyuk, O.; Butkiewicz, H.; Coleman, A. W.; Suwińska, K. Host-guest complexes of local anesthetics with cucurbit[6]uril and *para*-sulphonatocalix[8]arene in the solid state. *J. Mol. Struct.* **2017**, *1150*, 28-36.
10. Fang, H.; Li, G.; Jiang, D.; Zheng, G. A channel rotaxane coordination polymer (RCP) based on the assembly of *p*-sulfonatocalix[8]arene and 4,4′-bipyridine-N,N′-dioxide ligand. *Polyhedron* **2019**, *160*, 53-57.
11. Alex, J. M.; McArdle, P.; Crowley, P. B. Supramolecular stacking in a high Z′ calix[8]arene–porphyrin assembly. *CrystEngComm* **2020**, *22*, 14-17.
12. Kravets, K.; Kravets, M.; Kędra, K.; Danylyuk, O. *p*-Sulfonatocalix[8]arene coordinates sodium cations and forms host-guest complex with berberine: insight from crystal structure. *Supramol. Chem.* **2021**, *33*, 666-676.

13. Y. Zhao, D. G. Truhlar, The M06 suite of density functionals for main group thermochemistry, thermochemical kinetics, noncovalent interactions, excited states, and transition elements: two new functionals and systematic testing of four M06-class functionals and 12 other functionals. *Theor. Chem. Account* **2008**, *120*, 215-241.
14. M. J. Frisch, G. W. Trucks, H. B. Schlegel, G. E. Scuseria, M. A. Robb, J. R. Cheeseman, G. Scalmani, V. Barone, G. A. Petersson, H. Nakatsuji, X. Li, M. Caricato, A. V. Marenich, J. Bloino, B. G. Janesko, R. Gomperts, B. Mennucci, H. P. Hratchian, J. V. Ortiz, A. F. Izmaylov, J. L. Sonnenberg, D. Williams-Young, F. Ding, F. Lipparini, F. Egidi, J. Goings, B. Peng, A. Petrone, T. Henderson, D. Ranasinghe, V. G. Zakrzewski, J. Gao, N. Rega, G. Zheng, W. Liang, M. Hada, M. Ehara, K. Toyota, R. Fukuda, J. Hasegawa, M. Ishida, T. Nakajima, Y. Honda, O. Kitao, H. Nakai, T. Vreven, K. Throssell, J. A. Montgomery Jr., J. E. Peralta, F. Ogliaro, M. J. Bearpark, J. J. Heyd, E. N. Brothers, K. N. Kudin, V. N. Staroverov, T. A. Keith, R. Kobayashi, J. Normand, K. Raghavachari, A. P. Rendell, J. C. Burant, S. S. Iyengar, J. Tomasi, M. Cossi, J. M. Millam, M. Klene, C. Adamo, R. Cammi, J. W. Ochterski, R. L. Martin, K. Morokuma, O. Farkas, J. B. Foresman, D. J. Fox, *Gaussian 16 Revision C.01*, **2016**.
